# Shaping Future Generations of Public Health Researchers: *Preventing Chronic Disease*’s Student Research Paper Contest

**DOI:** 10.5888/pcd14.170431

**Published:** 2017-10-12

**Authors:** Leonard Jack

**Figure:**
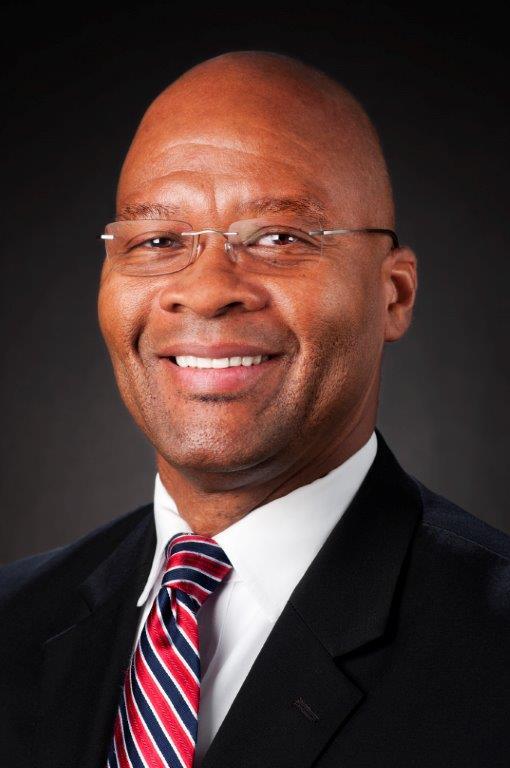
Leonard Jack, Jr, PhD, MSc, Editor in Chief

*Preventing Chronic Disease* (*PCD*) is committed to providing opportunities for future generations of researchers to contribute to public health and develop critical writing and reviewing skills. Since its introduction in 2011, *PCD*’s Student Research Paper Contest has been a success; each year the journal receives manuscripts prepared by students from around the world, and the number of entries continues to increase. This year, *PCD* set a record of 72 student submissions. With so many entries, we decided that the only fair way to judge the submissions would be to establish 4 winning categories by level of education: high school, undergraduate, graduate, and doctoral. This year’s submissions addressed a range of topics related to the screening, surveillance, and use of population-based approaches to prevent and control chronic diseases and focused on such health conditions as arthritis, asthma, cancer, diabetes, cardiovascular health, obesity, depression, and others.

## Goals of the *PCD* Student Research Paper Contest

There are 5 primary goals of *PCD*’s Student Research Paper Contest:

- Provide students with an opportunity to become familiar with a journal’s manuscript submission requirements and peer-review process
- Assist students to connect their knowledge and training on conducting quality research with a journal’s publication expectations
- Develop students’ research and scientific writing skills to become producers of knowledge rather than just consumers of knowledge
- Provide students with an opportunity to become a first author on a peer-reviewed article
- Promote supportive, respectful, and mutually beneficial student–mentor relationships that strengthen students’ ability to generate and submit scholarly manuscripts throughout their professional career

## Developing Critical Skills in Research and Scholarly Publishing

Conducting sound research and summarizing findings for a scholarly publication requires patience, resilience, sound ethical judgement, and scientific writing skills that meet a journal’s high standards. *PCD* recognizes that this process can be both an exciting and an anxiety-producing experience for student authors. *PCD* provides guidance and support through every stage of the publication process to help student authors gain experience and confidence. *PCD*’s website offers comprehensive guidance to authors in preparing manuscripts for submission, including helpful submission checklists, detailed descriptions of various article types and requirements, and guidelines on structuring abstracts and creating tables and figures from the *AMA Manual of Style: A Guide for Authors and Editors, 10th Edition* (1).

The contest also helps students during the early stages of their academic career in working with colleagues and responding to critique. In preparing their manuscripts, students have an opportunity to work with mentors to explore the public health landscape and identify original ideas that contribute to public health. The peer-review process student manuscripts undergo at *PCD* allows students to talk about appropriate ways to respond professionally to feedback. Novice authors can be discouraged by negative feedback, but through the peer review and revision process students learn the value of feedback in strengthening their arguments, clarifying their narrative story, and gaining knowledge and insight from peer reviewers who are subject matter experts in their field.

Another key aspect of the contest is helping students gain a greater understanding of the ethical parameters of peer-reviewed research, so that they develop good judgement in presenting and interpreting data. Students work with their mentors to better understand what it means to execute sound ethical judgement. *PCD* encourages and facilitates these conversations by providing guidance from the American Medical Association, the International Committee of Medical Journal Editors, and the Committee on Publication Ethics on topics such as duplicate publication, definitions of authorship, conflicts of interest, copyright and permissions, institutional review board approval, differences between honest scientific errors and research misconduct, and a detailed understanding of a peer-reviewed journal’s process for responding to allegations of possible misconduct.

And finally, every stage of *PCD*’s Student Research Paper Contest requires students to take responsibility in responding to deadlines. Students must submit their manuscript to *PCD* on or before the due date, respond to feedback from peer reviewers and the editor in chief, and work with *PCD*’s experienced staff of technical editors through all stages of editing and production. Manuscripts may undergo multiple rewrites as students respond to comments and suggestions related to the strengths and weaknesses of their study, statistical tests used, presentation of data in tables and figures, accuracy of data analyses, and implication of the study’s findings on public health research and practice. In advancing through these stages and meeting these deadlines, students develop two of the most critical skills of successful public health professionals: patience and persistence.

## Contest Categories and Stages of Review

This year’s winners in the high school, undergraduate, graduate, and doctoral categories should be commended for demonstrating maturity and professionalism throughout this comprehensive and intense manuscript submission and review process. *PCD*’s student papers progress through 6 stages. First, the editorial office screens entries to determine whether they meet contest requirements. In the second stage, the editor in chief reviews the entries to determine whether they align with the journal’s mission and vision and are of high enough quality to advance to the third stage. In the third stage, members of the *PCD* editorial board identify which submissions should be considered as potential winners for the various categories. Submissions not advancing as potential winners are assigned to *PCD*’s standard peer-review process, so that those students still have an opportunity for publication. In the fourth stage, the editorial board conducts a comprehensive review of a few selected manuscripts and provides feedback to the student contestants, who then must address the feedback and submit a revised manuscript. In the fifth stage, the editorial board assesses the revised manuscripts to identify which should be selected as the winner in each category. Editorial board members must provide strong justifications to support their selections to the editor in chief, who makes the final decision. The sixth and final stage is notifying authors of winners. In addition to having an article published, winning authors are featured through a *PCD* podcast, “*PCD* Sound Bites,” to discuss key aspects of their research. *PCD* also mentors winners by providing an opportunity for them to become a reviewer and serve on a selection panel for the next year’s contest.

## 2017 *PCD* Student Research Paper Contest Winners

*PCD* identified 5 winners in the 2017 Student Research Paper Contest. Two entries were selected as winners in the doctoral category. In one, Pacheco and colleagues conducted a study that followed a cohort of 673 participants in Chile from infancy to adolescence to understand the association between early obesity and risk of metabolic syndrome in adolescence (2). Researchers found that the age of onset of obesity is a strong risk factor for metabolic syndrome. In the other winning entry, Arlinghaus and colleagues conducted a 6-month obesity program for Hispanic middle school students in Houston, Texas, to determine the feasibility of using high school students as trained peer health mentors called *compañeros* to promote and sustain reductions in body mass index (3). Researchers found that the use of *compañeros* was a promising approach in helping Hispanic children achieve healthier body weight.

The winning entry in the graduate category, by Mendoza-Herrera and colleagues, described the development of a diabetic retinopathy screening tool based on a predictive model for use among low-income adults in Mexico (4). Researchers collected biomedical, clinical, anthropometric, and sociodemographic data from 1,000 low-income adults with diabetes. Four risk factors predicted diabetic retinopathy: time since diabetes diagnosis, hyperglycemia, systolic hypertension, and physical inactivity.

The winning entry in the undergraduate category, by Smurthwaite and Nasser, explored the geographic convergence of chronic conditions at the neighborhood level (5). The study used a cross-sectional design to estimate the prevalence of obesity, cardiovascular disease, and type 2 diabetes in western Adelaide, Australia. The authors used Moran’s *I* method to identify significant clusters of these 3 chronic conditions and observed diverse spatial variation in their prevalence.

*PCD* is delighted to have its first winner in the high school category. Liu and colleagues conducted a social marketing campaign that used environmental prompts to influence purchases of fruits and vegetables (6). The social marketing campaign was implemented and evaluated in 17 grocery stores during 4 months in 5 rural counties in Kentucky. By using surveys collected from 240 participants, the authors found that recipe cards influenced participants’ desire to purchase fruits and vegetables.

## Parting Thoughts

Students and mentors submitting manuscripts in this year’s Student Research Paper Contest — regardless of whether their entry was selected as a winner — should be proud of their efforts. Student authors of manuscripts not accepted for publication in *PCD* were encouraged to seek consideration elsewhere. *PCD* has just announced the call for student research papers for its 2018 contest. Please see our Announcements page (www.cdc.gov/pcd/announcements.htm) for more information. *PCD*’s Student Research Paper Contest has proven to be a well-received scientific writing experience. We ask *PCD* readers to encourage students to consider submitting a manuscript for consideration in next year’s contest.

## References

[1] Iverson C , Christiansen S , Flanagin A , Fontanarosa PB , Glass RM , Gregoline B , et al, editors. AMA manual of style: a guide for authors and editors, 10th edition. Oxford (UK): Oxford University Press; 2007.

[2] Pacheco LS , Blanco E , Burrows R , Reyes M , Lozoff B , Gahagan S . Early onset obesity and risk of metabolic syndrome among Chilean adolescents. Prev Chronic Dis 2017;14:170132. 10.5888/pcd14.170132 PMC5645192 29023232

[3] Arlinghaus KR , Moreno JP , Reesor L , Hernandez DC , Johnston CA . *Compañeros*: high school students mentor middle school students to address obesity among Hispanic adolescents. Prev Chronic Dis 2017;14:170130. 10.5888/pcd14.170130 PMC5645191 29023233

[4] Mendoza-Herrera K , Quezada AD , Pedroza-Tobia A , Hernández-Alcaraz C , Fromow-Guerra J , Barquera S . A diabetic retinopathy screening tool for low-income adults in Mexico. Prev Chronic Dis 2017;14:170157. 10.5888/pcd14.170157 PMC5645201 29023230

[5] Smurthwaite K , Bagheri N . Using geographical convergence of obesity, cardiovascular disease, and type 2 diabetes at the neighborhood level to inform policy and practice. Prev Chronic Dis 2017;14:170170. 10.5888/pcd14.170170 PMC5645193 29023234

[6] Liu E , Stephenson T , Houlihan J , Gustafson A . Marketing strategies to encourage rural residents of high-obesity counties to buy fruits and vegetables in grocery stores. Prev Chronic Dis 2017;14:170109. 10.5888/pcd14.170109 PMC5645197 29023231

